# Acute Stroke Care during COVID-19: National Data

**DOI:** 10.3390/idr14020024

**Published:** 2022-03-16

**Authors:** Grzegorz Miękisiak, Samuel D. Pettersson, Dariusz Szarek, Piotr Morasiewicz, Justyna Fercho, Stanisław Adamski, Lech Kipiński, Tomasz Szmuda

**Affiliations:** 1 Institute of Medicine, University of Opole, 45-040 Opole, Poland; piotr.morasiewicz@uni.opole.pl; 2 Department of Neurosurgery, Medical University of Gdansk, 80-210 Gdansk, Poland; samueldpettersson@gmail.com (S.D.P.); jfercho@uck.gda.pl (J.F.); tomasz.szmuda@gumed.edu.pl (T.S.); 3 Department of Neurosurgery, Marciniak’s Hospital, 54-049 Wrocław, Poland; szarekdariusz@gmail.com; 4 Neurosurgery Department, Copernicus Hospital, 80-152 Gdansk, Poland; stanislawadamski8@gmail.com; 5 Department of Pathophysiology, Wroclaw Medical University, 50-367 Wroclaw, Poland; lech.kipinski@umw.edu.pl

**Keywords:** acute stroke, COVID-19, pandemic

## Abstract

(1) Background: The pandemic of COVID-19 and subsequent lockdown strategies had a profound impact on many aspects of everyday life. During this time the world faced the unprecedented crisis of healthcare disrupting timely care delivery. This study was designed to evaluate the impact of the pandemic on the acute treatment of stroke in Poland. (2) Methods: The national data on hospitalizations with stroke as a primary diagnosis were obtained from the National Health Fund of Poland. Poisson regression was used to determine the significance of the change in hospital admissions. The differences between proportions were analyzed using the “N-1” Chi-squared test. (3) Results: During the COVID-19 period, the number of hospitalizations dropped by 8.28% with a monthly nadir of 22.02 in April. On a monthly scale during 2020, the greatest decrease was 22.02%. The thrombolysis ratio was also affected, with the highest monthly drop of 15.51% in November. The overall number of in-hospital deaths did not change. (4) Conclusions: The pandemic caused a serious disruption of the acute care of stroke. There is no evidence that the quality of care was seriously compromised.

## 1. Introduction

Stroke is the second leading cause of death and one of the major causes of permanent disability worldwide [1]. The annual incidence of stroke in the EU continues to rise, and Poland, despite efforts, has one of the highest incidence and mortality rates [2]. To properly handle patients experiencing the life threating condition, immediate evaluation, imaging, and initiation of reperfusion therapies are to be required on hand. If the immediate medical care is failed to be delivered in time, sever neurological complications or even death can occur. Therefore, the treatment of stroke requires multidisciplinary approaches which puts a tremendous strain on healthcare systems regardless of local socioeconomic conditions.

Poland is a medium-sized member country of the European Union with a population of 38.265 million [3]. It was affected by the COVID-19 pandemic as many other countries in the region with the first case of SARS-CoV-2 was registered on 4 March 2020 [4]. Soon later the restrictions were introduced abruptly and on March 12th, universities, schools, childcare providers, and culture institutions were closed [5]. A national state of emergency was declared on 14 March with additional restrictions implemented including having restaurants, bars, and cafes to operate only takeaways and delivery, banning of public gatherings of more than 50 people, and suspending all recreational or entertainment facilities. The borders were sealed the following day. All citizens were required to stay at home unless absolutely necessary while all international passenger air and rail systems were restricted. On 20 March, the prime minister announced the introduction of the epidemic state in Poland and the gradual lifting of the restrictions began on 20 April. The second wave hit the country in October, with a peak number of cases much higher than the first one. Restrictions were brought in again and remained in effect till the end of the year. According to official sources in 2020, there were 1,294,878 cases of SARS-CoV-2 with 28,554 confirmed deaths nationwide [6]. The national healthcare system became grossly overburdened in November that year. 

The global healthcare was put to the test as the pandemic was gaining momentum, overwhelming national health systems one after another. At the same time the human mobility was decreased to an unprecedented level. The combination of these and other factors have caused significant changes in the epidemiology of various conditions. The current literature contains multiple single and multicentric studies assessing the impact of the pandemic on stroke care. However, studies involving hospital databases carried out in a nationwide scale are rare and currently, the impact that the pandemic has had on the Polish Healthcare system is unknown. In the present study, we analyzed the national data on patients who received acute treatment for stroke. The effect of the pandemic was assessed by comparing the data on patients treated during the lockdown from March to December 2020 with the control group of patients who received treatment during the same months of the previous year. Overall, the findings from this study may be of great interest to healthcare authorities seeking to improve upon the current system.

## 2. Materials and Methods

The data were obtained from the National Health Fund of Poland (Narodowy Fundusz Zdrowia—NFZ), the main financing source of healthcare in the country. Since 2009, NFZ uses the diagnosis-related groups (DRGs) for reimbursement and regularly publishes comprehensive data on hospitalizations on an official website [4]. A monthly breakdown report was obtained from another government web platform “e-zdrowie” [7].

The data used in this study covered all hospitalizations with the stroke as a primary diagnosis during the period of 1 March–31 December 2020, as the first case of COVID-19 was diagnosed in Poland on 4 March 2020. The analogous period of 2019 was used as a control. The following DRGs were analyzed: A48—complex treatment of stroke in stroke units for 7+ days, A49—treatment of stroke for 3+ days, and A50—other treatment of stroke. The above groups covered all relevant hospitalizations nationwide in the specified period.

Poisson regression was used to determine the significance of the change in hospital admissions. The differences between proportions were analyzed using the “N-1” Chi-squared test. Statistical analyses were carried out using StatsDirect version 3.3.5 (StatsDirect Ltd., Merseyside, UK; http://www.statsdirect.com/ accessed on 24 October 2021) and MedCalc v. 12.5.0.0 (MedCalc Software, Ostend, Belgium).

## 3. Results

**Figure 1:**
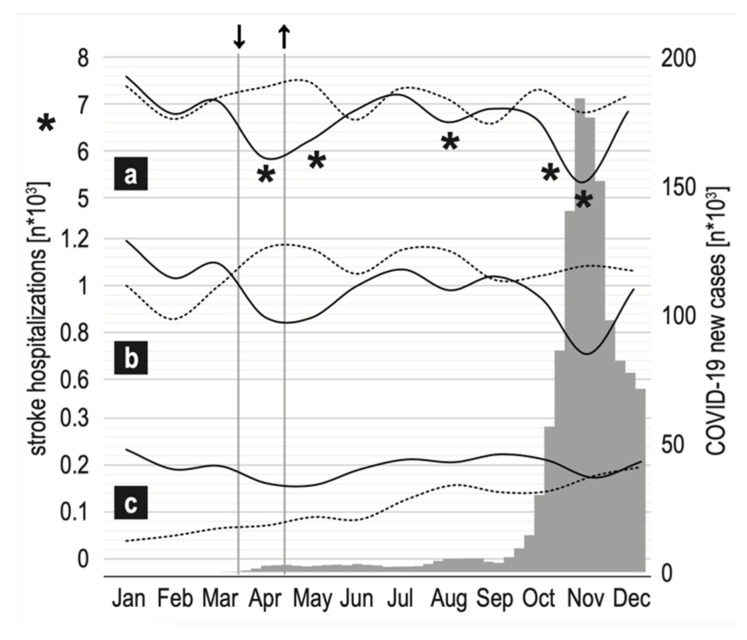
The timeline of the COVID-19 pandemic. (**a**) all stroke hospitalizations, (**b**) hospitalizations w/thrombolysis, (**c**) hospitalizations w/mechanical thrombectomy. Solid line2019, dotted line—2020, grey bars—new COVID-19 cases, arr↓—the movement restrictions introduced, ↑—the movement restriction lifted. * *p* < 0.0001.

During the COVID-19 period, the overall number of hospitalizations dropped by 8.28% compared with the respective period of 2019 (Table 1). On a monthly scale of the 2020, the greatest decrease being 22.02% was measured in April and the second, at 21.08% in November. The decrease was statistically significant in April, May, August, October, and November as per Poisson regression model (Figure 1).

## 4. Discussion

The present study is based on the national data obtained from the National Health Fund, covering all hospitalizations due to stroke in the country during the last 10 months of 2019 and 2020. The data revealed that the number of hospitalizations due to stroke during the pandemic dropped by 8.28% compared with the respective time period of 2019, with biggest drops in April (22.02%) and November (21.08%). Similar findings were reported by authors throughout the world [5,8,9,10] with a drop of weekly admissions as high as 39.52% during the critical period [10]. In our material, two nadirs were noted during the COVID moths: the first one in April and the second in November. The former was most likely caused by the combination of fear of unfamiliar disease which may have prevented people from seeking medical attention on top of the movement restrictions which were introduced by the government on 25 March 2020 [11]. Under the new regulations, no one could leave their homes “without rational excuse”. This restriction which was most severe thus far, was lifted on 20 April. The second nadir correlated with the second wave peak of the pandemic which occurred on 7 November [12]. The decrease in the number of hospitalizations cannot be explained with the changes in incidence but rather due to poor access to acute health services. In April it was caused by the combination of restrictions and the fear of contracting the COVID-19 in a hospital. Whereas in November, the access to emergency medical services was severely restricted as the national healthcare was grossly overburdened. Nioi et al. [13] in their institutional experience in Norther Italy report similar conclusions. They mention that during Italy’s hard lockdown period (March–May 2020), patients’ fear associated with contacting the virus led to a lower request for healthcare services and hospitalizations. Additionally, Perry et al. [14] in a recent study found that most of the pandemic decline in stroke was among those with the mildest form. Firstly, these are the patients who are most likely to manage stroke at home and secondly, the disease can be more easily overlooked in ERs which are working at the maximum capacity. In Poland we witnessed both developments, the former in April and the latter in November. This conclusion has a potentially grave consequences as approximately 10% of these patients will have a recurrent stroke within weeks [15]. A significant increase of the in-hospital mortality rate (8.80%) with the steady number of deaths observed in this study is consistent with the above rationale that more severe strokes were finding their way to stroke units. There was a significant decrease in the thrombolysis rate, from the mean of 17.97% during the period III–XII 2019 to 17.15% during the analogous time frame. The lowest ratio was noted in November: 12.6%, a drop of 15.51% compared with November 2019. The reperfusion therapy was affected in many other reports worldwide, with the drop of thrombolysis ratio ranging from 20.9% in France [16] to 26.7% in China [17]. Various plausible explanations have been proposed, such as patients’ late arrivals to the hospital, lengthening of intrahospital delays, and physicians’ preference for primary thrombectomy [18,19]. The changes in the pattern of mechanical thrombectomy apparent in this study was a result of a recent introduction of a national pilot program of mechanical thrombectomy. With the program starting at the end of 2018 [20], a meaningful comparison is not possible. The demographics were also affected by the lockdown. The male to female ratio was increased by from 0.98 to 1.05. The authors of another study from Netherlands [5], also noticed changes in the gender pattern, although in their case more women sought medical attention. They hypothesized that the threshold to wait out the stroke symptoms at home was higher for men. The differences in our data are likely due to cultural differences. We have found differences in the age of hospitalized patients. Most notably, there was significantly less senile patients, aged 80+. This was likely caused by the even more limited mobility in this age group, which was considered most vulnerable from the early days of pandemic. Many nursing homes were shut, and the staff was often neglected by health authorities [21], leaving them helpless in the face of the crisis.

Aside from assessing how the pandemic has impacted the statistics regarding patient demographics and stroke characteristics of those admitted, it is also important to address how patient recovery has been affected by the current crisis. Unfortunately, detailed follow up information is not reported by the National Health Fund of Poland and therefore, this could not be assessed by the present study. However, one multicentric study from China has assessed patient recovery from stroke while utilizing a quality-of-life survey taken by patients during their recovery in the hospital [22]. Surprisingly, the quality-of-life score was found to be significantly higher during the pandemic when compared to the pre-pandemic period. The authors proposed that the strict implemented regulations on patient visits, allocation of non-infectious patients in separate rooms, and new disinfection protocols are to be the cause. The result of the implemented protocols which were introduced in the early stages of the pandemic resulted in a more isolated and a safer environment for the noninfectious patients. However, it is also worth assessing patient recovery from stroke after hospital discharge in the home setting. A recent study from the United Kingdom assessing patient reported health outcomes after hospitalization with stroke taken at 30-day follow-up identified the pandemic period as independently associated with a poorer health outcome compared to the pre-pandemic period [23]. The cause of the lower heath outcome is speculated to be due the behavioral factors induced by the pandemic such as: decrease in community care; a lack of informal social support; reduced monitoring of severe symptoms in the community; lack of direct links to primary care; increased loneliness; the challenges of living in confined households; limited availability of remote healthcare interventions; and increased anxiety about the trajectory of stroke recovery. Despite only one study currently having assessed the quality-of-life of patients after being discharged, based on the patients from the study, it is clear that the current care is inadequate. Greater psychological support and an increase in the availability of specialists via telecommunications for the patients recovering at home is of great need. The findings from the two studies mentioned are merely a crude insight into how the pandemic is impacting the quality of recovery from stroke. Given that no national study currently exists on assessing the quality of the recovery from stroke during the pandemic, we encourage future national studies to assess this factor.

Lastly, it is critical to mention ways to improve upon the current protocol implemented by the nation’s healthcare system for treating stroke patients in similar crises. The first wave of COVID-19 caused a great anxiety among patients and medical personnel causing a significant disruption of healthcare. However, as the medical community learned more about the disease the system was soon brought back to nearly full capacity. The second gridlock in the treatment of stroke and other emergencies was caused by the overwhelming effect of the large volume of COVID-19 patients the entire health system. In order to optimize treatment in case of similar occurrences in the future both issues listed above should be addressed with new recommendations, put forth on a national level. The implementation of such new protocols among hospitals is known to be especially difficult [24,25,26,27,28]. In Poland, all public hospitals are owned independently by the local government in order to maintain hospital managerial autonomy. However, with changes needing to be approved by each local authority, this decentralized method of hospital management results in hospitals allocating resources and approving new protocols asynchronously unlike in centralized management. Thus, it is known that reforms implemented by the central government in the past were often not accompanied by appropriate management changes at the level of individual hospitals. This is mainly due to lack of cooperation between the hospital owners and the inability of the hospitals to break-even due to cost increases driven mainly by external factors such as regulatory salary increases [29]. Therefore, greater influence of centralized hospital decision making is needed during similar crises. Aside from the financial and political aspects, a reorganization of the stroke-care network with promotion of the mothership paradigm would be of great interest; the development of new in-hospital care pathways based on continuous analysis of local data; and nationwide education campaigns about the importance of immediate response to stroke and TIA symptoms. Moreover, a campaign is required to reassure patients that hospitals provide safe in-person medical care in cases of alarming symptoms [29].

There are several limitations to this study. Firstly, the Polish national database does not report individual patient by listing each individual’s diagnoses, complications, etc. Therefore, only a univariate analysis was possible to be performed. A multivariate analysis to identify if our significant results are as of direct result of the COVID-19 pandemic is thus not possible to be performed. Secondly, the data extracted covered all hospitalizations with stroke as a primary diagnosis. However, due to COVID-19 being a prothrombotic disease [30], a risk of bias may be present in our results regarding thrombotic rates as we were not able to identify which patients reported during the pandemic period for stroke additionally developed COVID nosocomially. Logically, it would be suspected that these patients, co-diagnosed with stroke and COVID, would contribute to an increase thrombotic rate in the year 2020. Surprisingly, was not the case as we identified a significant decrease in the rate of thrombosis. Our hypothesis is that the lack of seeking medical attention among individuals due to the fear of obtaining COVID at hospitals has outweighed the effects of COVID potentially increasing thrombotic rates due to being a pro-thrombotic disease.

## 5. Conclusions

This study represents data on all strokes in Poland. Our results identified that the pandemic caused a serious disruption of the acute care of stroke with a decrease in hospitalizations as low as 22.02% during April. Although there is no evidence that the quality of care was seriously compromised, our findings suggest that new protocols must be implemented into hospitals nationwide which improve the availability of stroke treatment during extraordinary times. With multiple studies from various countries reporting the similar findings of significant decreased hospitalizations during the pandemic period, it is likely that patients with risk of stroke are affected by the pandemic at a global level. Given that the end of the COVID-19 pandemic is unable to be speculated as new strains are being identified, healthcare protocols which are meticulously designed to hedge against crisis affecting the healthcare system are greatly needed.

## Figures and Tables

**Table 1:** Comparison of groups between the pre-COVID-19 period and 2020 involving hospitalizations for stroke. * *p* < 0.05, ** *p* < 0.001.

	Mar–Dec 2020		Mar–Dec 2019	
General Stats				
total patients [n(per annum)]	64,129		69,379	
total hospitalizations [n(per annum)]	65,520		70,943	
Demographics: sex gender				
F:M ratio	1.05 *		0.98 *	
Demographics: age				
Age group	n	%	n	%
<1	6	0.01	8	0.01
1–6	24	0.04	12	0.02
7–17	37	0.06	44	0.06
18–40	1251	1.95	1305	1.88
41–60	9282	14.47	10,206	14.71
61–80	35,315	55.07 *	37,520	54.08 *
80+	18,214	28.4*	20,281	29.23 *
Thrombolytic therapy				
Thrombolysis: total	9677		10,896	
% of all	17.15 *		17.97 *	
Mechanical thrombectomy	1928		1242	
% of all	3.01 **		1.79 **	
Diagnoses				
Diagnosis (ICD10)	n	%	n	%
Ischemic stroke (I63)	56,436	88	60,641	87.41
Hemorrhagic stroke (I61)	4004	6.24	4458	6.43
other stroke	3689	5.75	4280	6.17
Mortality				
in-house deaths [n(per annum)]	10,229		10,172	
mortality rate [%]	15.95 **		14.66 **	

## Data Availability

Not applicable.
